# Viral Infection Identifies Micropeptides Differentially Regulated in smORF-Containing lncRNAs

**DOI:** 10.3390/genes8080206

**Published:** 2017-08-21

**Authors:** Brandon S. Razooky, Benedikt Obermayer, Joshua Biggs O’May, Alexander Tarakhovsky

**Affiliations:** 1 Laboratory of Immune Cell Epigenetics and Signaling, The Rockefeller University, New York, NY 10065, USA; brazooky@gmail.com; 2 Laboratory of Virology and Infectious Diseases, The Rockefeller University, New York, NY 10065, USA; 3 Berlin Institute for Medical Systems Biology, Max Delbrück Center for Molecular Medicine, 13125 Berlin, Germany; benedikt.obermayer@mdc-berlin.de

**Keywords:** small open reading frames, micropeptides, ribosome profiling, viral infection

## Abstract

Viral infection leads to a robust cellular response whereby the infected cell produces hundreds of molecular regulators to combat infection. Currently, non-canonical components, e.g., long noncoding RNAs (lncRNAs) have been added to the repertoire of immune regulators involved in the antiviral program. Interestingly, studies utilizing next-generation sequencing technologies show that a subset of the >10,000 lncRNAs in the mammalian genome contain small open reading frames (smORFs) associated with active translation, i.e., many lncRNAs are not noncoding. Here, we use genome-wide high-throughput methods to identify potential micropeptides in smORF-containing lncRNAs involved in the immune response. Using influenza as a viral infection model, we performed RNA-seq and ribosome profiling to track expression and translation of putative lncRNAs that may encode for peptides and identify tens of potential candidates. Interestingly, many of these peptides are highly conserved at the protein level, strongly suggesting biological relevance and activity. By perusing publicly available data sets, four potential peptides of interest seem common to stress induction and/or are highly conserved; potential peptides from the MMP24-AS1, ZFAS1, RP11-622K12.1, and MIR22HG genes. Interestingly, using an antibody against the potential peptide encoded by MIR22HG RNA, we show that the peptide is stably expressed in the absence of infection, and upregulated in response to infection, corroborating the prediction of the ribosome profiling results. These data show the utility of perturbation approaches in identifying potentially relevant novel molecules encoded in the genome.

## 1. Introduction

The host/parasite relationship between cells and viruses epitomizes the Red Queen Hypothesis, i.e., constant coevolution between competing organisms just to remain extant [1]. Thus, it is not surprising that cells evolved intricate systems to combat viruses. In vertebrates, cells suppress viral replication through the following programs: (*i*) an intrinsic component that inhibits intracellular viral replication within an isolated cell [2], (*ii*) an innate system that inhibits intracellular viral replication and sends extracellular warning signals to cells which can lead to activation of the third category of antiviral activity, (*iii*) the adaptive response which prevents further viral dissemination and leads to organism-level memory formation [3,4].

The immune programs laying the lines of defense against viral invasion are composed of a diverse array of small molecule products and macromolecular components (ranging from RNA and proteins to lipids). Interestingly, recent advances in genome-wide technologies have highlighted that the catalog of molecules regulating and involved in the antiviral response is incomplete [5]. For example, a class of RNAs termed long noncoding RNAs (lncRNAs)—lncRNAs are >200 base pairs and subject to many of the same post-transcriptional modifications as mRNAs—has been identified as key players of antiviral regulation, despite not serving as templates for translation [6,7,8,9]. These lncRNAs can act as scaffolds to bring protein complexes together, regulate protein translocation, as well as host and viral gene expression [8,10,11].

What is striking is that many lncRNAs seem to be misclassified as non-coding, as a fraction of the >10,000 lncRNAs do in fact harbor small open reading frames (smORFs) [12,13,14]. These smORF-containing lncRNAs would potentially encode for micropeptides, i.e., peptides <100 amino acids in length [15]. Recent advances in proteomics and genome-wide technologies, specifically ribosome profiling, have facilitated the discovery of this previously unknown pool of smORFs encoding for micropeptides in lncRNAs [16]. Whether every smORF generates a biologically relevant protein product is still under scrutiny; however, in a few striking examples, conserved smORFs in lncRNAs were translated into functional micropeptides [17]. For example, a few micropeptides were found to regulate Ca^2+^ flux in cardiac [18] and muscle cells [19], and are conserved across mammals [20]. Notably, these micropeptides differ significantly from classically defined bioactive peptides as micropeptides in lncRNAs seem to lack many of the salient features of the latter, such as secretion signals or cleavage from larger proteins [21]. In light of these findings, it is apparent that there is a fundamental knowledge gap regarding the role of smORFs and the micropeptides they encode in regulating cellular behavior [22].

Here, we aim to uncover novel peptide-based regulators of the antiviral response hidden amongst the slew of lncRNAs in the cellular genome. Relying on the broad cellular responses upon infection and using a combination of next-generation sequencing methodologies and biochemical approaches, we identify tens of novel smORF-containing lncRNAs differentially regulated upon infection. The data reveal the kinetic properties of lncRNA expression and translation upon viral infection. Many of the micropeptides can be stably expressed, and for one, the endogenous protein levels were probed and matched the upregulation upon infection observed by ribosome profiling techniques. These descriptive studies on novel micropeptides shed light on how our understanding of antiviral expression and the coordinated program remains incomplete.

## 2. Materials and Methods

### 2.1. Ribosome Profiling and RNA-seq Library Preparation 

For ribosome profiling experiments, A549 cells were grown in a 15 cm dish in culture medium. Cells were infected with PR8 or PR8ΔNS1 as described [23]. Control cells were not subject to any media changes, while mock infected cells were washed with phosphate-buffered saline (PBS), rocked in 4 mL of Dulbecco’s Modified Eagle’s medium (DMEM) without any supplements for 1 h, then washed with PBS, followed by the addition of culture medium. Twelve hours post infection, cells were harvested and library preparation proceeded according to the manufacturer’s protocol using the TruSeq Ribo Profile for Mammalian kit (Illumina^®^, Carlsbad, CA, USA). A list of additional reagents can be found within the manufacturer’s protocol and were used as described within. Depletion of ribosomal RNA was performed using the Ribo-Zero rRNA Removal Kit (H/M/R) (Illumina^®^). Sequencing libraries were pooled together in groups of four per lane and sequenced at the Genome Resource Center at The Rockefeller University using the Illumina HiSeq 2500, 50 cycle SR_Multiplexed. Sequencing data have been deposited in NCBI’s Gene Expression Omnibus and are accessible through GEO Series accession number GSE101760.

### 2.2. High-Throughput Sequencing Analysis 

Raw reads were subjected to three successive rounds of adapter trimming using Flexbar [24] with the adapter sequence AGATCGGAAGAGCACACGTCT. We then depleted ribosomal RNA using bowtie2 by mapping against a database of human ribosomal RNA obtained from SILVA [25] and RefSeq [26]. The remaining (non-ribosomal) reads were then jointly mapped against the human (hg19) and viral (PR8 H1N1) genomes using STAR and the Gencode v19 annotation. Gene expression was quantified for RNA using featureCounts [27] and the Gencode v19 annotation. In parallel, we used the ORFscore method [13] on the pooled ribosome protected fragment (RPF) reads to detect translated ORFs (AUG-stop) in the human transcriptome. After inspection of metagene plots generated by custom scripts, we used read lengths of 27 to 31 nt and defined P-sites as 12 nt downstream of the 5′ end. We then used a cutoff of 6 on the ORFscore, required at least 5% of codons to be covered and less than 30% of used reads mapping to multiple loci. The translated ORFs defined in this way for each gene were transformed into simplified annotation format (SAF) format to serve as reference for the quantification of RPF reads using featureCounts. We then estimated log2 fold changes (PR8 vs. mock and PR8dNS1 vs. mock) for the RNA and RPF data separately using DESeq2 [28] and the total number of mapped reads (human + viral) to normalize libraries. Changes in translational efficiency (TE) were estimated from RPF data using RNA as the covariate. Genes with translated smORFs were defined as those with all translated ORFs smaller than 100 aa (using ORFscore, aggregating all transcript isoforms, and disregarding smORFs in 3′ or 5′ UTRs or in alternative reading frames) and distinguished by gene biotype. Conservation scores on the nucleotide (phyloP) [29] and protein level (phyloCSF) [30], respectively, were extracted based on our previously published pipeline (micPDP) [31]. Gene ontology (GO) term analysis was performed using GOAtools [32,33], comparing genes with absolute log2 fold change >1 on the RPF level against all translated genes as a background, and selecting categories with at least 5 genes and corrected *p*-value < 0.01. For the re-analysis of the data of Tirosh et al., we downloaded reads from GEO (accession GSE69906) and processed them with the same pipeline.

### 2.3. Quantitative PCR Methods 

Cells were infected at an MOI of 1 with PR8 or PR8ΔNS1 strains (along with control and mock infected cells). The 10^6^ cells were collected and lysed using the QIAshredder kit (Qiagen, Hilden, Germany). RNA was DNase treated using an RNase free DNase set (Qiagen) and cellular RNA was extracted using the RNeasy kit (Qiagen) and quantified using a Nanodrop (ThermoFisher, Waltham, MA, USA). An amount of 1 μg of RNA from each cell line was reverse transcribed into cDNA, using the Transcriptor First Strand cDNA Synthesis Kit (Roche, Basel, Switzerland) utilizing both poly dT and random hexamers. cDNA was analyzed using the SYBR Green Master Mix (Roche) in 384-well plate format in a LightCycler^®^ 480 Instrument (Roche) using a standard protocol. Primer sequences are available upon request. All quantitative PCR (qPCR) results with influenza infection were normalized to actin as previously described [23].

**Protein analysis.** Purified CD4 T cells were lysed with 0.5% NP-40 containing TMSD lysis buffer (40 mM Tris-HCl pH 7.9, 5 mM MgCl_2_, 250 mM Sucrose, 1 mM DTT, 0.5% NP-40 (IGEPAL), protease cocktails (Sigma Aldrich, St. Louis, MO, USA), phosphatase cocktails (Millipore, Billerica, MA, USA) on ice for 15 min. Protein concentrations were determined by standard BCA assay. The extracts or whole cell lysates were separated by SDS-PAGE and transferred to a polyvinylidene difluoride membrane. The membrane was blocked, incubated with primary antibodies, followed by horseradish peroxidases-conjugated secondary antibodies and developed with ECL systems according to the manufacturer’s instructions. The antibodies used were as follows: MIR22HG Anti-C17orf91 antibody-C-terminal (ab140282, Abcam, Cambridge, UK).

### 2.4. Cell and Viral Culture 

A549 cells were obtained from ATCC (ATCC^®^ CCL-185^TM^). A549 and 293FT cells were passaged and cultured in DMEM + l-glutamine, 10% FBS, 1% PenStrep at 37 degrees Celsius, and 5% CO_2_ in humidified conditions. PR8 and PR8ΔNS1 stocks were produced and titered as previously described [34]. Single-round infection assays on A549 cells was performed by infecting cells with PR8 or PR8ΔNS1. Cells were then fixed using the Foxp3/Transcription Factor Staining Buffer Set (Affymetrix, Santa Clara, CA, USA) according to the manufacturer’s protocol. Cells were stained for nucleoprotein using anti-influenza A virus nucleoprotein antibody [431] (FITC) (ab20921) (Abcam), then taken to the flow cytometer for measurement.

### 2.5. Cloning 

The predicted sequence for the candidate micropeptides was ascertained as described in the high-throughput sequencing analysis section. For each peptide, the RNA sequence was codon optimized (Table S1), then the gene fragment was synthesized using IDT GeneBlocks (Coralville, IA, USA). Each GeneBlock was digested with BamH1/Not1 and cloned into a similarly cut pLenti-(multiple-cloning site)-IRES-GFP backbone.

### 2.6. Flow Cytometry 

Flow data were obtained on a BD LSRII instrument. At least one thousand events were collected per sample. Flow cytometry analysis was performed using FlowJo^©^ analysis software.

## 3. Results

**Figure 1:**
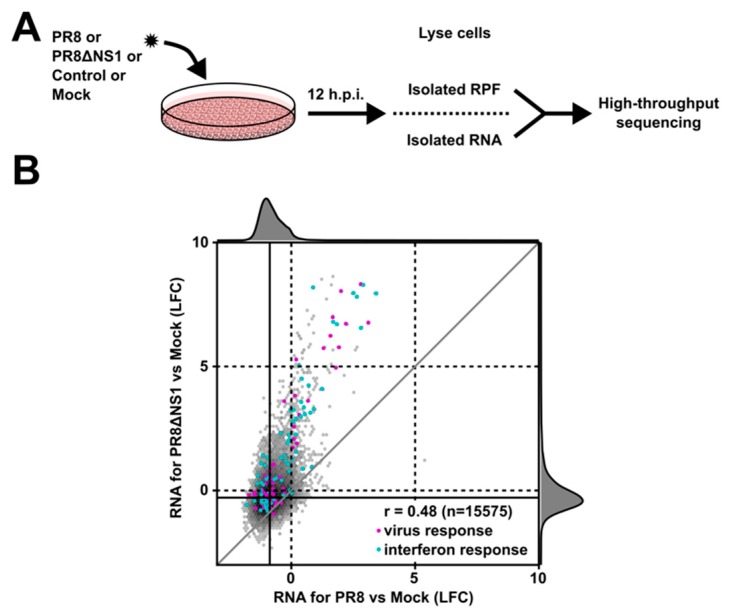
Identification of small open reading frame (smORF)-containing long noncoding RNAs (lncRNAs) differentially regulated upon infection. (**A**) Experimental scheme to identify novel micropeptides. A549 cells were infected with either influenza PR8 or PR8ΔNS1. Twelve hours post infection, cells were collected, and ribosome-protected RNA (RPF) and total RNA was isolated and sequenced; (**B**) Comparison of log2 fold changes in PR8 and PR8ΔNS1 infected cells compared to mock infection. Genes associated with virus and interferon response gene ontology (GO) terms are highlighted in purple and light blue, respectively. h.p.i.: hours post infection; LFC: log2 fold change.

To identify novel micropeptides involved in viral replication and inhibition, A549 cells were infected with well-characterized flu isolates; either wild-type influenza A strain H1N1 PR8, which encodes for a potent suppressor of innate immunity genes, or a derivative virus, PR8ΔNS1, which is immune-activating [23]. PR8 robustly inhibits antiviral gene expression through expression of the non-structural 1 (NS1) protein which inhibits the signaling cascade leading to interferon production. In contrast, the PR8ΔNS1 strain—harboring a large deletion that removes the majority of NS1—generates robust activation of the cellular antiviral response, interferon secretion, and clearance of the virus from even basic culture systems [23]. The utility of infecting with these two viruses is to perform differential analysis on lncRNAs significantly up- or downregulated during infection with PR8 and PR8ΔNS1 versus mock infected and control cells (Figure 1A). As these two viruses differ in level of induced antiviral response, purely virus-induced (infection with PR8) versus (infection with PR8ΔNS1) antiviral-induced lncRNAs and potential micropeptides can be identified and scored.

We chose to perform the profiling experiment twelve hours post infection as pilot experiments with PR8ΔNS1 show a robust antiviral response at this time point (Figure S1). Cells were infected at an MOI of 1 with PR8, PR8ΔNS1, remained uninfected (“mock”) or completely unperturbed. The computational analysis focused on identifying novel smORFs that are significantly up/down-regulated during infection. To determine this, we used DESeq2 [28] to estimate log2 fold changes between infected and uninfected cells on the level of RNA, RPF or translational efficiency (TE), normalizing libraries to the total number of mapped reads (human and viral genome) to account for virus-induced host shutoff effects [35]. Next, to detect translated smORFs in a lncRNA, we used ORFscore [13] to inspect ribosome profiling reads mapping to the human transcriptome for signatures of significant triplet phasing, which is indicative of active translation. We finally also scored the level of sequence conservation on the nucleotide (phyloP) or protein level (phyloCSF) as done previously [31]. Collectively, these metrics allow comparison between the control, mock, PR8 and PR8ΔNS1 libraries to test for lncRNAs and smORFs differentially expressed and/or translated (Figures S2–S7). One caveat of this approach is that potential micropeptides not differentially expressed, but instead alternatively regulated, e.g., by post-translational modifications that regulate activity, will be overlooked. However, using only the differential expression analysis (Figure 1B) and RPF read phasing analysis as metrics, 19 candidate smORFs in lncRNAs or other noncoding transcripts (among 52 novel smORFs in total) were identified that are up- or downregulated more than twofold compared to the response of the entire transcriptome (median log fold change between infection with PR8ΔNS1 or PR8 and control or mock infected cells) (Table S1). More generally, we found 3 of 13 annotated lncRNAs containing novel smORFs differentially regulated (23%), while 98% of expressed lncRNAs (1903 of 1938) remained unchanged, indicating that translated lncRNAs are more likely to be regulated under infection than non-translated lncRNAs (*p* = 0.0026 by Fisher’s exact test).

Beyond the scope of smORFs, infection with these flu isolates showed dramatic changes in the entire transcriptome, while mock infection did not trigger any changes compared to untreated cells (Figure S2). Upon infection with the PR8 strain, we observed host shutoff with an associated takeover of the RNA and protein synthesis machinery by the virus, indicated by ~50% and ~30% of RNA and RPF reads, respectively, mapping to the viral transcriptome (Figures S4 and S7) [35]. In contrast, this effect is strongly attenuated in the PR8dNS1 infection (Figure S3). The massive changes in transcription and translation are in agreement with previous analyses of cells exposed to alternate immune-triggering stimuli such as human cytomegalovirus (HCMV) (Figure S9) [36].

**Figure 2:**
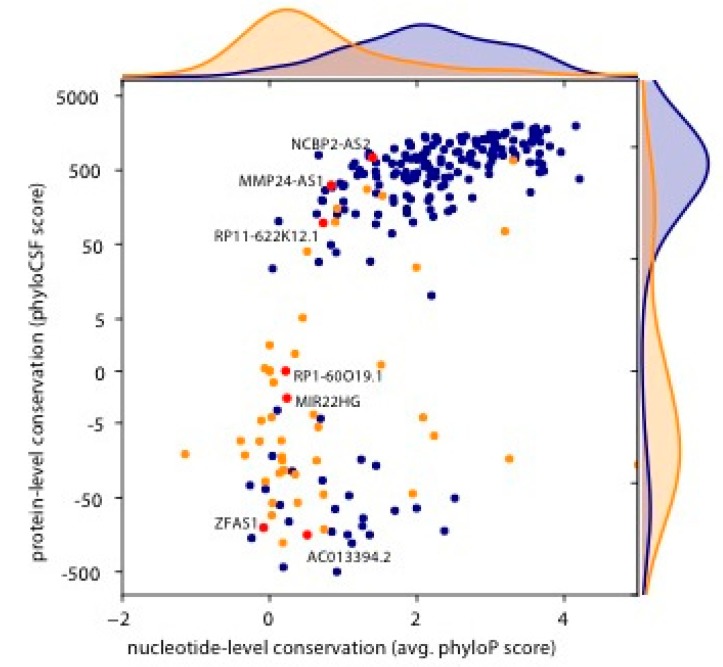
Conservation of nucleotide and amino acid sequence of the smORFs within lncRNAs identified upon viral infection. Plot of the conservation of the amino acid and nucleotide sequence of the candidate micropeptides encoded by lncRNAs. The phyloCSF score (y-axis) is a measure of amino acid conservation and the phyloP score (x-axis) indicates the nucleotide level conservation. The candidate smORFs (red dots) vary widely in amino acid and nucleotide level conservation. Blue dots indicate annotated ORFs, while orange dots indicate novel smORFs.

Interestingly, some of the nucleotide and amino acid sequences of the potential micropeptides in the identified smORFs are highly conserved (Figure 2). We assessed nucleotide sequence conservation using phyloP [29] and amino acid sequence conservation with phyloCSF [30], which scores the depletion of nonsynonymous codon substitutions expected from coding regions. For annotated smORFs, the two metrics are strongly correlated (Spearman’s rho = 0.69), i.e., translated regions that are more constrained on the nucleotide level generally show selective constraint also on the encoded amino acid sequence. Novel smORFs are generally much less conserved at both the nucleotide and amino acid sequence level (*p* < 10^−13^ by a Mann–Whitney test in both cases); nevertheless, we observe a weak correlation between the two metrics (*rho* = 0.30, *p* = 0.03). While some novel candidates, notably MMP24-AS1 and RP11-622K12.1/LINC01420/NoBody [37], exhibit the conservation hallmarks of genuine protein-coding genes, many others do not show preferential conservation of their (nucleotide or amino acid) sequence.

**Figure 3:**
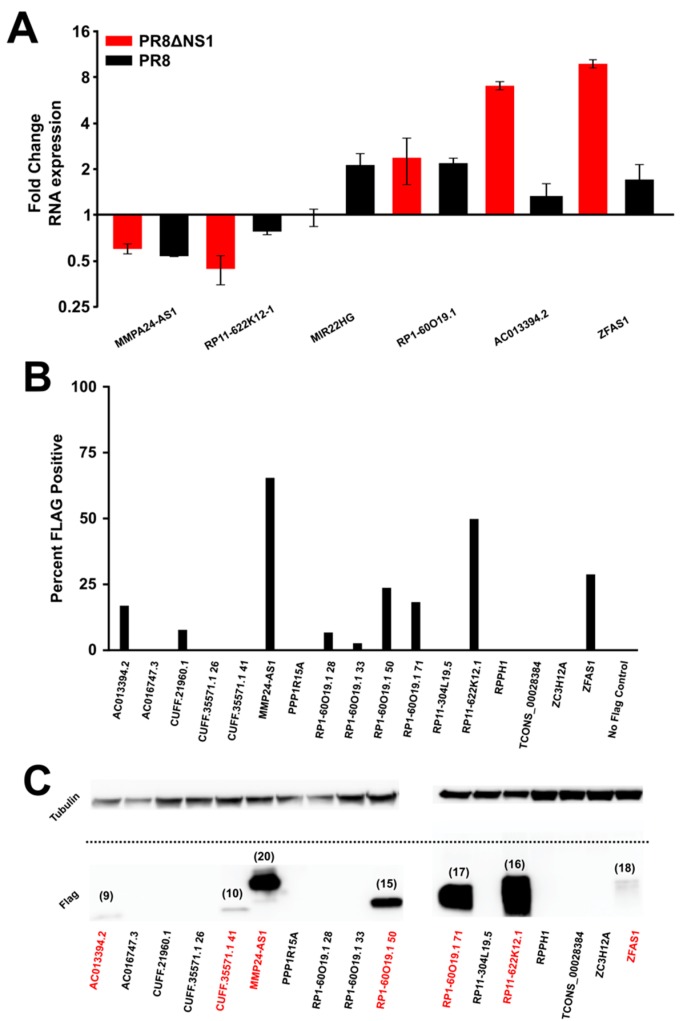
A subset of the micropeptides can be ectopically expressed upon transient transfection. (**A**) qPCR results for various lncRNAs corroborate the findings of RNA-seq. Error bars represent the standard deviation from three independent experiments (Table S2). All data were normalized to actin. (**B**) Bar graphs representing the percent of cells expressing FLAG peptide or GFP upon transient transfection. GFP expression indicates that the mRNA is produced and translated, while the FLAG signal represents the percent of cells with detectable levels of micropeptide. (**C**) Protein blot analysis shows that a subset of the micropeptides can be stably expressed and the band sizes (numbers above the bands, in kD) are in line with estimates from the ribosome profiling data (Table S1).

To ascertain the validity of the findings in the RNA-seq data, qPCR primers were developed against selected lncRNAs. At 12 h post infection, qPCR was performed to assay the levels of particular lncRNAs in response to infection with PR8 or PR8dNS1 (Figure 3A). Importantly, the general trend of up- or down-regulation of the lncRNAs observed in the qPCR data (Figure 3A) matches the results observed for RNA-seq (Table S2). Collectively, these data verify smORF-containing lncRNA expression changes in response to viral infection and innate immunity triggering. Next, expression plasmids encoding for the predicted micropeptide sequences and containing a GFP reporter were utilized to test for the stability and ability of these peptides to be expressed. The micropeptide was tagged with a FLAG sequence to allow for rapid detection by flow cytometry and Western blotting. Of a panel of tested smORF-encoded micropeptides transiently transfected into 293FT cells (Table S1), despite robust GFP expression from each plasmid, only a subset of the peptides could be detected by flow cytometry (Figure 3B). Notably, many of the highly conserved micropeptides such as MMP24-AS1 and RP11-622K12.1 were highly expressed, while those less conserved, such as AC013394.2 compared to ZFAS1, were expressed to variable levels (Figure 2 and Figure 3B). As GFP is expressed from the same mRNA, these data suggest that either the ribosome skips over the peptide sequence, or some of the peptides are inherently unstable. The latter seems likely as the expression plasmid is the same for each peptide, and some of the peptides can be expressed quite stably. To be certain that the correct peptides were detected by flow cytometry, Western blot analysis was performed. Corroborating the results from the flow cytometry, a fraction of the peptides were expressed at detectable levels, and, importantly, the detected the bands ran at the predicted sizes (Figure 3C and Figure S8, and Table S1). Of the products that do show some stability, it would be of interest to identify their potential role in cellular function, or more specifically, during the course of viral infection.

**Figure 4:**
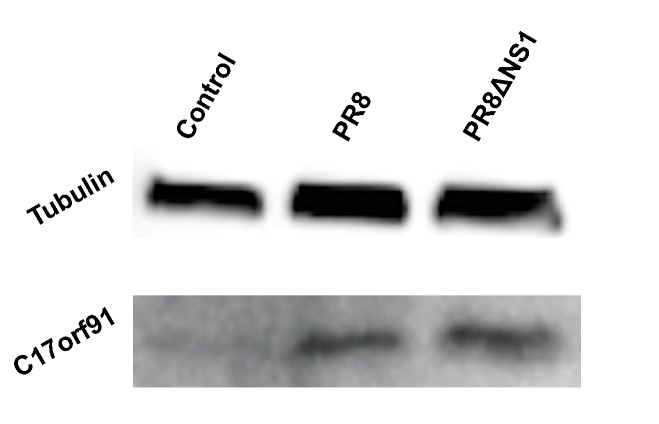
The endogenous micropeptide encoded by MIR22HG is upregulated upon infection. Western blot of MIR22HG peptide upon infection. The MIR22HG peptide is detectable in the absence of infection, and upon infection with PR8 or PR8ΔNS1 at a MOI of 1 the expression level increases. MOI: multiplicity of infection.

The data indicate that a large fraction of lncRNAs are differentially regulated upon infection. The biological function of these peptides remains outside the scope of this study, but the fact that stable peptides are generated as a result of infection is particularly interesting. To test this directly, A549 cells were infected at an MOI of 1 and probed for a particular peptide generated from a specific lncRNA: MIR22HG, the host gene for miR-22. Previous work has shown that MIR22HG is upregulated in response to chemical stresses [38,39]. Beyond these studies, not much is known about the role of this lncRNA and we were unable to find publicly available information on the peptide that it may potentially encode. This predicted peptide is ~9 kD, and Western blot analysis of control cells as well as cells infected with either PR8 or PR8ΔNS1 indeed show upregulation of the endogenous peptide (Figure 4 and Figure S10). The upregulation of the lncRNA seems specific to PR8 infection (Figure 3), thus it is surprising to see the peptide upregulated upon infection with PR8ΔNS1 (Figure 4). As the PR8 strain of influenza is a potent inhibitor of innate immune responses, these data suggest that the MIR22HG peptide is either stabilized, occurring independently of innate signaling, but in response to cellular stress most likely induced by viral infection. This would also explain why the peptide is also shown to increase upon infection with PR8ΔNS1.

## 4. Discussion

Current findings are steadily adding to our understanding of the complexity of genomes, epitomized by new studies on aspects of transcription, translation, and various macromolecular modifications [16,40]. High-throughput and high read depth technologies are essential to tabulating these novel, biologically relevant products. Here, a combination of RNA-seq and ribosome profiling identified the complex transcriptional and translational changes occurring upon infection of a host cell with influenza. Previously, to identify potential peptides, conservation analysis was performed [13,20]. Here, we provide a perturbation approach, coupling ribosome profiling and influenza infection, to search for micropeptides encoded in lncRNAs. Our focus on the differential regulation of lncRNAs with translated smORFs adds to the growing list of biologically relevant products [15].

Interestingly, more conserved peptides were more readily expressed, while less conserved peptides had variable expression capabilities. Specifically, the peptides encoded by the lncRNAs MMP24-AS1 and RP11-622K12.1 [37] were readily expressed to high levels, and these amino acid sequences are highly conserved (Figure 2). As previous analysis relied on conservation scores to identify bioactive peptides [13,20], these data corroborate that the conservation of particular amino acid sequences is in fact informative for peptide expression. The other micropeptides that were not highly conserved were highly variable in the ability to express; for example, the peptides encoded by the lncRNAs RP1-60O19.1, ZFAS1, AC013394.2 (Figure 3A). The lack of predictability in expression for nonconserved sequences suggests that each peptide identified would require individual experimentation in a high-throughput manner to assess biological relevance. At this point, we tried to express 17 different potential peptides, and seven were readily expressed (Figure 3B), but this work only presents a subset of the smORFs in the genome. Importantly, these micropeptides were also chosen through an indirect method, ribosome profiling and analysis of periodicity plots (Figure S6), suggesting more direct methods such as mass spectrometry may aid in identifying peptides present in a cell. In the future, protein expression libraries or CRISPR/Cas knockout screens should focus on these peptides to identify their biological relevance.

Finally, we were able to detect an endogenous cellular-generated peptide (Figure 4), corroborating increased peptide expression from the MIR22HG lncRNA predicted from ribosome profiling and RNA-seq data (Figure 1). Currently, there is a lack of resources for detecting endogenous peptides, though efforts are underway to develop tools and analyses to detect and probe their function [40]. As new studies focus on micropeptides [41,42], these concerted efforts will help to understand the role of micropeptides to regulate immunity and general biological function.

## Supplementary Materials

The following are available online at www.mdpi.com/2073-4425/8/8/206/s1. Figure S1: PR8 suppresses innate immune activation, while PR8∆NS1 leads to a robust interferon response as measured by qPCR; Figure S2: Mock and control cells do not show any significant changes in gene expression as measured by RNAseq and ribosome profiling; Figure S3: Comparison of PR8∆NS1 RNAseq and ribosome profiling experiments; Figure S4: Comparison of duplicate PR8 RNAseq and ribosome profiling experiments; Figure S5: Gene ontology (GO) analysis of genes upregulated in RNAseq and ribosome profiling experiments in cells infected with either PR8 or PR8∆NS1; Figure S6: Periodicity plots show that the identified smORFs are likely translated; Figure S7: PR8 leads to host shutoff of transcription and translation; Figure S8: Raw image of ectopically expressed micropeptides; Figure S9: Reanalysis of publically available data shows that interferon stimulation of cells or infection with HCMV leads to similar large changes in transcription and translation; Figure S10: Raw image of western probing for MIR22HG; Table S1: List of smORFs encoded in lncRNAs identified upon viral infection, smORF sequences used in Figure 3 and the predicted sizes; Table S2: Comparison of RNAseq and qPCR expression.
