# Supplementary material for: Viral Infection Identifies Micropeptides Differentially Regulated in smORF-Containing lncRNAs
Source: Genes (Basel). 2017 Aug 21;8(8):206. doi: 10.3390/genes8080206 (PMC5575669; doi:10.3390/genes8080206)
Supplement: Supplementary file 1 [file genes-08-00206-s001.zip › SI_Material/20170814_SI_BR.docx]

**Supplemental Information for:**

**Viral infection identifies micropeptides differentially regulated in smORF-containing lncRNA**

Brandon S. Razooky^1,2,§^, Benedikt Obermayer^3,§^, Joshua Biggs-O’May^4^, Alexander Tarakhovsky^1,*^

The file includes:

Figures S1-S10

Table of Contents

Supplementary Figures3

Figure S1. PR8 suppresses innate immune activation, while PR8∆NS1 leads to a robust interferon response as measured by qPCR3

Figure S2. Mock and control cells do not show any significant changes in gene expression as measured by RNAseq and ribosome profiling4

Figure S3. Comparison of PR8∆NS1 RNAseq and ribosome profiling experiments5

Figure S4. Comparison of PR8 RNAseq and ribosome profiling experiments6

Figure S5. Gene ontology (GO) analysis of genes upregulated in RNAseq and ribosome profiling experiments in cells infected with either PR8 or PR8∆NS17

Figure S6. Periodicity plots show that the identified smORFs are likely translated8

Figure S7. PR8 leads to host shutoff of transcription and translation9

Figure S8. Raw image of ectopically expressed micropeptides with FLAG tag10

Figure S9. Reanalysis of publically available data shows that interferon stimulation of cells or infection with HCMV leads to similar large changes in transcription and translation11

Figure S10. Raw image of western probing for MIR22HG12

**Figure S1. PR8 suppresses innate immune activation, while PR8∆NS1 leads to a robust interferon response as measured by qPCR.** Delta-delta Ct plot. qPCR primers were developed to probe for levels of nucleoprotein (flu_NP), non-structural protein 1 (flu_PR8), interferon-beta (IFNB), and retinoic-acid inducible gene I (RIG-I) twelve hours post infection with influenza PR8 or influenza PR8∆NS1 at an MOI of 1. The measurements were normalized to actin.

**Figure S2. Mock and control cells do not show any significant changes in gene expression as measured by RNAseq and ribosome profiling.** RPF control vs mock and RNA control vs mock experiments show no significant changes in virus response (purple dots) or interferon response (blue dots) or all 8933 genes. Virus and interferon response genes were categorized by gene ontology analysis (see materials and methods). The histograms on the outside of the plots show the distribution in ribosome profiling and RNAseq reads.

**Figure S3. Comparison of PR8∆NS1 RNAseq and ribosome profiling experiments.** Comparison of RPF and RNA measurements for PR8∆NS1 infection shows a high correlation between differential regulation of mRNA reads and ribosome reads (for 8904 genes). Blue dots indicate annotated smORFs previously identified in the literature, orange dots represent novel smORFs identified in this study, and red dots represent candidate smORFs that are the focus of this study. The histograms on the outside of the plots show the distribution in ribosome profiling and RNAseq reads. Dotted lines indicate median LFC values across all genes.

**Figure S4. Comparison of duplicate PR8 RNAseq and ribosome profiling experiments.** Comparison of RPF and RNA measurements for PR8∆NS1 infection shows a high correlation between differential regulation of mRNA reads and ribosome reads (for 8931 genes). Blue dots indicate annotated smORFs previously identified in the literature, orange dots represent novel smORFs identified in this study, and red dots represent candidate smORFs that are the focus of this study. The histograms on the outside of the plots show the distribution in ribosome profiling and RNAseq reads. Dotted lines indicate median LFC values across all genes.

**Figure S5. Gene ontology (GO) analysis of genes upregulated in RNAseq and ribosome profiling experiments in cells infected with either PR8 or PR8∆NS1.** GO analysis upon PR8 and PR8**∆**NS1 infection ascertained from the RNAseq and ribosome profiling experiments. Many of the GO terms are involved in immunity, inflammation, cellular stress responses, and general viral inhibition pathways. As described in the main text, PR8**∆**NS1 is a potent activator of innate immunity, while PR8 infection suppresses immune responses.

**Figure S6. Periodicity plots show that the identified smORFs are likely translated.** The plots show the fraction of reads that align to the first, second or third frame of the RNA sequence. The first frame is identified as the start codon with the most reads that is also in frame with a stop codon.

**Figure S7. PR8 leads to host shutoff of transcription and translation.** A large fraction of the reads in RNAseq or RPF reads in PR8 infected cells are for the virus. The large changes in transcriptional and translational reads upon PR8 infection suggest a takeover of host resources, an ability lost in the PR8∆NS1.


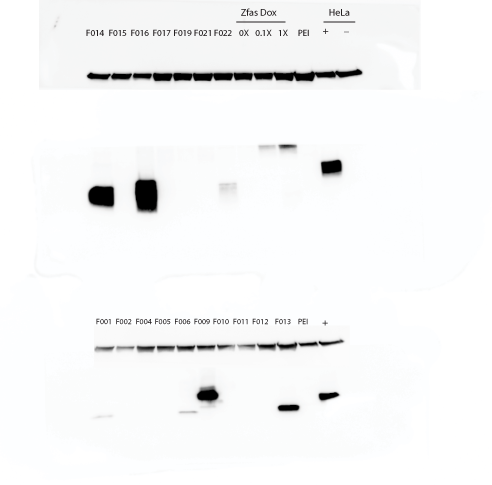

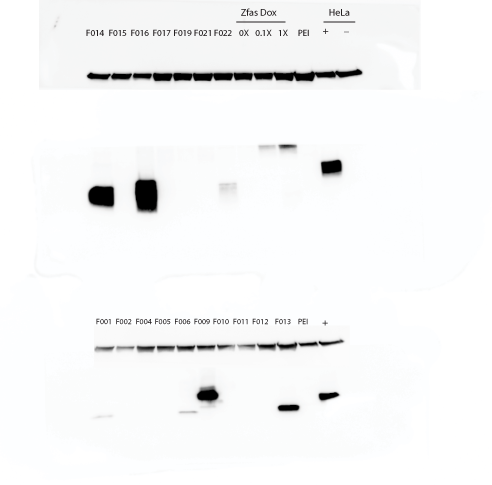


**Figure S8. Raw image of ectopically expressed micropeptides with FLAG tag.** These images were used to create panel C in Figure 3 of the main text. The labels for the lanes with expression correspond to the following: F001 (AC013394.2), F006 (CUFF.35571.1 41), F009 (MMP24-AS1), F013 (RP1-60O19.1 50), + (), F014 (RP1-60O19.1 71), F016 (RP11-622K12.1), F022 (ZFAS1), Zfas Dox 0x, 0.1x, 1x (ZFAS1-mRuby), HeLa + (). The HeLa +, bottom panel, and +, top panel, labels correspond to histone H3.3 expressed from a doxycycline inducible promoter where the + means dox was added to the cells. The H3.3 is fused to a FLAG-HA tag expected to run at around 20kD. The Zfas Dox is a fusion between ZFAS peptide and a fluorescent protein, expected to run at around 30kD and also expressed from a doxycycline inducible promoter. These raw images are provided for a reference point for the size of the micropeptides in Figure 3.

**Figure S9. Reanalysis of publically available data shows that interferon stimulation of cells or infection with HCMV leads to similar large changes in transcription and translation.** Scatter plots of log2 fold changes in HCMV infected or IFN stimulated cells versus PR8 or PR8ΔNS1 cells for RNAseq and ribosome profiling reads. Virus and interferon stimulated genes and lncRNA/micropeptides identified in this study are highlighted in magenta, cyan and red, respectively, and show the similarity between HCMV infection, IFN stimulation and influenza infection. Correlation values (Spearman's rho) are taken across all expressed or translated genes as indicated in the plots.


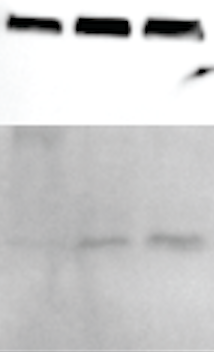


**Figure S10. Raw image of western probing for MIR22HG.** Top panel is Tubulin and bottom panel is mir22HG. The expanded view of the bottom panel shows the specificity and the band is running around the expected size of 8kD. The left bands are mock infected, middle is PR8delNS1 infection and right is PR8 infection. For more information on the antibody used to detect this band see http://www.abcam.com/c17orf91-antibody-c-terminal-ab140282.html.
